# Transcriptome Sequencing and Differential Analysis of Testes in One- and Two-Year-Old Kazakh Horses

**DOI:** 10.3390/ani16081220

**Published:** 2026-04-16

**Authors:** Yi Su, Liuxiang Wen, Jiaqi Jiang, Mingyue Wen, Yaqi Zeng, Jun Meng, Jianwen Wang, Wanlu Ren, Xinkui Yao

**Affiliations:** 1 College of Animal Science, Xinjiang Agricultural University, Urumqi 830052, China; 13335339131@163.com (Y.S.); 13890451520@163.com (L.W.); 13779313490@163.com (J.J.); 18280584310@163.com (M.W.); zengyaqi@xjau.edu.cn (Y.Z.); mengjun@xjau.edu.cn (J.M.); dkwjw@xjau.edu.cn (J.W.); 2 Xinjiang Key Laboratory of Equine Breeding and Exercise Physiology, Urumqi 830052, China

**Keywords:** Kazakh horse, testis, differentially expressed genes, transcriptomics

## Abstract

This study systematically elucidated the developmental characteristics and molecular regulatory mechanisms of the testis during the critical period of sexual maturation in Kazakh horses by combining histological observation of one- and two-year-old testicular tissues with transcriptomic sequencing. In the testes of one-year-old horses, no obvious lumen was observed, and the interior is mainly comprising supporting cells and spermatogonia on the basement membrane; in contrast, in the testes of two-year-old horses, the tubular lumen was complete with spermatogonia, spermatocytes, and spermatozoa, indicating that spermatogenic function had approached maturity. Transcriptome profiling identified 979 differentially expressed genes (DEGs), with 209 up-regulated genes, including *CYP11A1* and *CATSPER2*, and 770 down-regulated genes, including *CD9*. Gene Ontology (GO) annotation indicated primary enrichment of DEGs in biological processes related to multicellular organism development, cell membrane composition, and ion binding. Kyoto Encyclopedia of Genes and Genomes (KEGG) pathway analysis showed significant enrichment of DEGs in the calcium signaling pathway, cell adhesion molecules, and neuroactive ligand–receptor interaction, among other key pathways. Protein–protein interaction (PPI) network analysis further highlighted core genes, including *TNF*, *CATSPER2*, and *CDH13*. Validation by RT-qPCR confirmed the reliability of the RNA-Seq data. Our findings reveal the dynamics of testicular development in Kazakh horses through histological and molecular analyses, thereby providing a theoretical framework and candidate genes to further elucidate regulatory mechanisms and guide genetic improvement in reproductive traits.

## Simple Summary

This study, by combining histological observation and transcriptome sequencing, systematically reveals the developmental characteristics and molecular regulatory mechanisms of the testes of one- and two-year-old Kazakh horses during the critical period of sexual maturity. In the testes of one-year-old horses, histological analysis found no obvious lumen, and the interior mainly comprised supporting cells and spermatogonia on the basement membrane. In the testes of two-year-old horses, the lumina were complete, and spermatogonia, spermatocytes, and sperm could be observed, indicating that spermatogenic function was approaching maturity. Functional enrichment analysis indicated biological processes, including multicellular organism development and cell membrane structural composition, related to the differentially expressed genes and significant enrichment of key pathways, including calcium signaling and cell adhesion molecules. The protein interaction network identified core genes, including *TNF* and *CATSPER2*. These findings elucidate the dynamic changes in testicular development at both the tissue and molecular levels, providing theoretical references and candidate genes for genetic improvement related to reproductive performance in Kazakh horses.

## 1. Introduction

Kazakh horses are an excellent indigenous breed in China, primarily distributed in northern Xinjiang. They exhibit notable advantages of tolerance to coarse feed and strong disease resistance [1,2]. Kazakh horses reach sexual maturity at two to three years, exhibit seasonal estrus, and show stable reproductive performance [3]. With the introduction of superior foreign breeds such as purebred horses and Orlov horses, the development of domestic local breeds has become increasingly important. Kazakh horses are versatile animals for both riding and draft purposes [4] and possess moderate meat and milk production capacities, with a substantial supply of mare’s milk and horse meat [5,6]. Enhancing the reproductive performance of Kazakh horses is a critical focus of contemporary breed development.

The testes, as complex and essential organs within the male reproductive system of vertebrates, are crucial in spermatogenesis and hormone secretion [7,8]. Testicular tissue comprises seminiferous tubules and interstitial tissue, containing diverse cell types including germ cells, supporting (Sertoli) cells, and interstitial (Leydig) cells [9,10]. Their development involves coordinated processes of cell division, migration, and differentiation orchestrated by multiple endocrine, paracrine, and intracellular signaling pathways. Testis development crucially underlies the reproductive capacity of stallions [11,12]. During equine growth, the seminiferous tubules are formed by one year of age, although germ cell development remains incomplete and the interstitial cell population is abundant [13,14]. By two years of age, under the influence of the gonads, the testes undergo rapid growth, with markedly increasing diameter and cross-sectional area of the seminiferous tubules, which facilitates germ cell formation and storage and ensures proper sperm transport [15,16].

Although morphological studies have described the developmental process of the testes, they fall short when elucidating the underlying molecular regulatory mechanisms. In recent years, rapid advancements in high-throughput transcriptome sequencing technologies have provided new methodological approaches for detecting gene expression levels holistically and identifying key functional genes [17,18]. By comparing transcriptomes of tissues or cells at different developmental stages or under various physiological conditions, the identified differentially expressed genes (DEGs) can be systematically screened. Subsequently, through gene ontology (GO) functional annotation and Kyoto Encyclopedia of Genes and Genomes (KEGG) pathway enrichment analysis, key genes and their associated regulatory pathways involved in specific biological processes can be identified [19]. For instance, Liu et al. [14] analyzed the testicular transcriptome of Mongolian horses before and after sexual maturity. They found that genes in the sexually immature stage were mainly enriched in pathways related to cellular infrastructure, whereas genes in the sexually mature stage were enriched in pathways associated with hormones, metabolism, and spermatogenesis. Shen et al. [20] conducted a whole-transcriptome m6A analysis of Xia’nan cattle testes across three developmental stages and identified the involvement of *PLK4*, *PTEN*, *EGR1*, and *PSME4* in the regulation of mammalian testicular development and spermatogenesis. These studies provide new insights into mammalian testicular development. However, research on testicular development during mammalian sexual maturation remains limited. Consequently, a comparative analysis of the gene expression profiles of the testes in one- and two-year-old Kazakh horses holds significant scientific value for elucidating the molecular regulatory mechanisms underlying their testicular development.

This study utilized the testicular tissues of healthy Kazakh horses at one and two years of age. Through histomorphological observation and RNA-sequencing analysis, it aimed to provide a theoretical foundation for a deeper understanding of testicular development in Kazakh horses and identify important candidate genes for the genetic improvement of their reproductive performance.

## 2. Materials and Methods

### 2.1. Experimental Animals and Sample Collection

Eight Kazakh horses (four each aged one and two years) from Wantong Animal Husbandry Technology Co., Ltd. in Emin County, Tacheng region, Xinjiang, China, were included. Testicular tissue samples were collected immediately after slaughter and fixed in a 4% paraformaldehyde solution (Servicebio, Wuhan, China) for histochemical analysis and stored in liquid nitrogen for subsequent transcriptomic studies.

### 2.2. Histochemical Characterization of the Testicular Tissue

Testicular samples fixed with paraformaldehyde were dehydrated through an ascending concentration gradient of alcohol (75%, 85%, 90%, 95%, and 100%) and turned transparent in xylene. The tissues were embedded in paraffin and sectioned into 5-μm-thick cross-sections using a microtome. The sections were stained with hematoxylin and eosin (H&E). After staining, the testicular tissue sections were examined and imaged under an optical microscope (Eclipse E100, Nikon, Shinagawa, Japan) equipped with a digital photography system.

### 2.3. Transcriptome Sequencing

Total RNA was extracted from testicular tissues using the TRIzol reagent (Invitrogen, Carlsbad, CA, USA) following the manufacturer’s instructions. The RNA quality was assessed by measuring the A260/A280 and A260/A230 ratios on a NanoDrop 2000 spectrophotometer (Thermo Fisher Scientific, Waltham, MA, USA). RNA integrity was verified by Agilent 2100 Bioanalyzer (Agilent Technologies, Santa Clara, CA, USA), and all samples had an RNA Integrity Number (RIN) greater than 8.0. After purification, RNA was fragmented and reverse-transcribed into corresponding cDNA to construct a strand-specific transcriptome library using the Hieff NGS^®^ Ultima Dual-mode mRNA Library Prep Kit (Yeasen, Shanghai, China). Sequencing was performed on the Illumina NovaSeq 6000 platform (Repugene Technology, Hangzhou, China) by Hangzhou Astrocyte Technology Co., Ltd., yielding 150 bp paired-end reads [21].

### 2.4. Data Quality Control

Raw data in FASTQ format obtained directly from the sequencer contained reads with adapters and low-quality reads. These raw reads were processed using Fastp (version 0.23.2) to remove adapter sequences and low-quality bases with the following filtering parameters: A sliding window of 4 bp with an average quality threshold of Q20 and a minimum read length of 50 bp after trimming [22]. The resulting clean reads were used for subsequent analysis. Metrics of the cleaned data, including Q20, Q30, and GC content, were calculated.

### 2.5. Reference Genome Alignment

Quality-controlled clean reads were aligned to the horse reference genome (EquCab3.0) using HISAT2 (version 2.2.1). The number of reads mapped to each gene was counted using featureCounts (version 2.0.3, from the Subread package). Gene expression was normalized and expressed as Fragments Per Kilobase of transcript per Million mapped reads (FPKM).

### 2.6. Differential Expression Analysis

Differential gene expression (DEG) analysis was performed using the DESeq2 R package (version 1.40.2). Genes with an adjusted *p*-value (False Discovery Rate, FDR) <0.05 and an absolute log2(fold change) > 1 were considered as significantly differentially expressed.

### 2.7. GO and KEGG Enrichment Analysis

Enrichment analysis was conducted for differentially expressed mRNAs, using GO and KEGG pathway analyses for annotation. The KOBAS software (version 3.0) was used to test the statistical enrichment of KEGG pathways related to DEGs; GO functional analysis was performed using the GOseq software (version 1.56.0). The threshold for significant enrichment was set at *p* < 0.05.

### 2.8. Protein–Protein Interaction (PPI) Network Analysis

The PPI of DEGs was constructed using the STRING database (version 12.0, https://string-db.org, accessed on 28 November 2025). The minimum required interaction score confidence threshold was set to >0.70 and included known ortholog interactions. In Cytoscape (version 3.10.0), the “Betweenness-unDir” plugin in the CytoNCA module was utilized to filter for central targets and visually analyze the results.

### 2.9. RT-qPCR Validation

Eight randomly selected differentially expressed mRNAs were subjected to real-time quantitative PCR (qPCR) to validate the transcriptome sequencing results. Total RNA was extracted from testis tissue using RNA extraction reagent (Servicebio, G3013, Wuhan, China). Approximately 20 mg of tissue was added to 1 mL of RNA extraction reagent and thoroughly ground in a three-dimensional cryogenic grinder. Subsequently, 200 μL of chloroform substitute was added, mixed well, and centrifuged at 12,000 rpm for 10 min at 4 °C. The supernatant was collected. RNA was precipitated using an equal volume of isopropanol, washed with 75% ethanol, and dissolved in nuclease-free water. RNA concentration and purity were determined on the NanoDrop 2000 spectrophotometer, with A260/A280 ratios between 1.8 and 2.0. One microgram of total RNA was reverse transcribed in a 20 μL reaction system using the SweScript All-in-One RT SuperMix for qPCR kit (Servicebio, G3330, Wuhan, China), with the program set to 25 °C for 5 min, 42 °C for 30 min, and 85 °C for 5 s. Amplification was carried out using 2X Universal Blue SYBR Green qPCR Master Mix (Servicebio, Q511, Wuhan, China) on a CFX Connect real-time fluorescence quantitative PCR system (Bio-Rad, Hercules, CA, USA). The 20 μL reaction system included 7.5 μL of premix, 0.25 μM forward and reverse primers (sequences shown in Table S1), and 1 μL of diluted cDNA template. The amplification program was as follows: pre-denaturation at 95 °C for 30 s, followed by 40 cycles of 95 °C for 5 s and 60 °C for 30 s. The melt curve analysis was conducted from 65 °C to 95 °C, collecting fluorescence signals for every 0.5 °C increase. All samples were run in triplicate. Relative gene expression was calculated using the 2^(−ΔΔCt) method, with GAPDH and β-actin serving as internal reference genes for normalization.

## 3. Results and Analysis

### 3.1. Histochemical Characteristics of the Testicular Tissue

**Figure 1:**
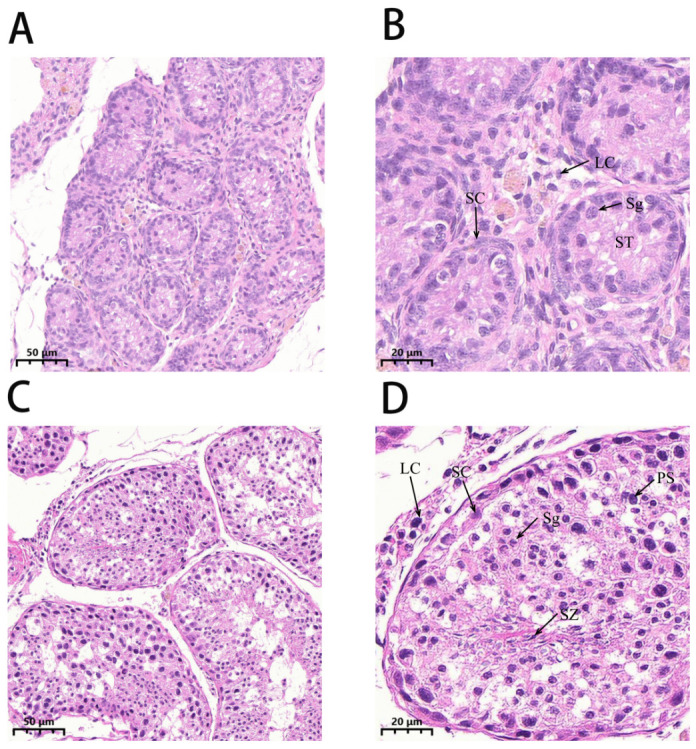
Hematoxylin-Eosin staining of testicular tissues from Kazakh horses of different ages. Note: ST: Seminiferous tubules; SC: Supporting cells; Sg: Spermatogonia; LC: Interstitial cells; PS: Primary spermatocytes; SZ: Sperm; circle indicates cellular vacuolization. (**A**) Testis of a one-year-old Kazakh horse (20×). (**B**) Testis of a one-year-old Kazakh horse (50×). (**C**) Testis of a two-year-old Kazakh horse (20×). (**D**) Testis of a two-year-old Kazakh horse (50×).

After HE staining, the seminiferous tubules of the one-year-old Kazakh horse testes exhibited a relatively small diameter, with no lumen formation. The seminiferous tubules contained only Sertoli cells and spermatogonia, closely arranged on the basement membrane in a single-layered structure. A large number of Leydig cells were distributed around the seminiferous tubules (Figure 1A,B).

In the testes of two-year-old Kazakh horses, the diameter of the seminiferous tubules increased, and the lumen structure was clearly defined. The seminiferous epithelium appeared intact, with normal germ cell morphology. From the outer to the inner layers, Sertoli cells, spermatogonia, spermatocytes, round and elongated spermatids, and spermatozoa were sequentially observed (Figure 1C,D).

### 3.2. Sequencing Quality Analysis

Testicular tissue samples from one- and two-year-old horses were collected, and a total of eight transcriptome samples were sequenced. After quality control, a total of 99,358,832,661 clean reads were obtained (average: 12,419,854,082.625 per sample). The GC content ranged between 49.67% and 51.67%, consistent with the expected base composition pattern. Both Q20 and Q30 scores were above 98% and 94%, respectively, indicating high data reliability (Table 1). When aligning the clean data to the reference genome, over 94.02% of the data were accurately mapped, demonstrating a high alignment rate (Table 2). The sequencing qualification rates for both Group G-1 and Group G-2 exceeded 90%. Thus, transcriptome sequencing data were of high quality and suitable for analysis.

### 3.3. Inter-Sample Correlation Analysis

**Figure 2:**
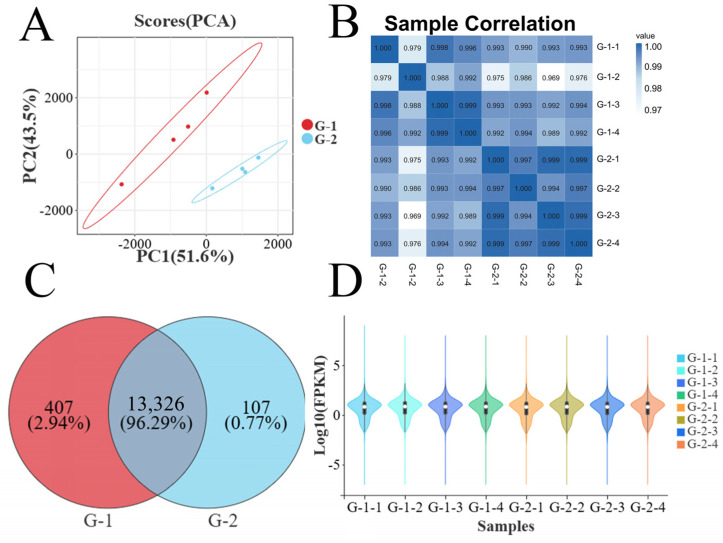
PCA and Sample Correlation Heatmap for Group G-1 and Group G-2. (**A**) Principal Component Analysis (PCA) plot for genes in Group G1 and Group G2; (**B**) Heatmap of correlation analysis for genes on Group G1 and Group G2. (**C**) Venn diagram for genes common to Group G1 and Group G2; (**D**) Violin plot of genes in Group G1 and Group G2.

Principal Component Analysis (PCA) of testicular samples from Kazakh horses of different ages is shown in Figure 2A. The first principal component (PC1), representing the primary coordinate, contributed 51.6% of the total variation, while the second principal component (PC2), as the secondary coordinate, accounted for 43.5%. The cluster distribution between groups was relatively loose, indicating significant heterogeneity within each group. As illustrated in Figure 2C, 13,733 and 13,433 genes were identified in the testicular tissue from one- and two-year-old groups, respectively, with 13,326 genes common to both groups. Furthermore, correlations among samples across groups exhibited similar trends (Figure 2B,D).

### 3.4. Screening DEGs

**Figure 3:**
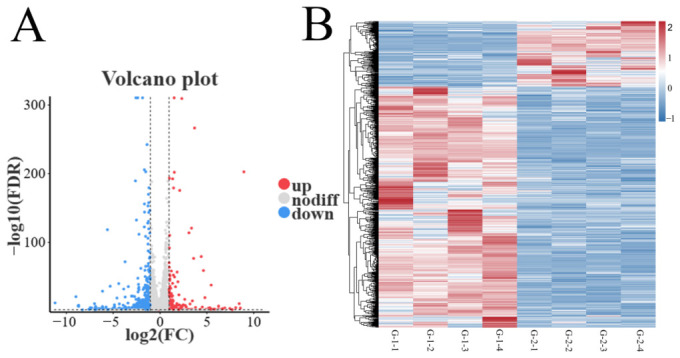
Volcano plot and cluster analysis of DEGs. (**A**) Volcano plot for genes in Group G1 vs. Group G2; (**B**) Cluster analysis for genes in Group G1 vs. Group G2.

According to the results of differential expression analysis of testicular tissues from Kazakh horses of different ages, as shown in Figure 3A, 979 DEGs, including *HNF4G*, *GAD2*, *FGD1*, *SLITRK3*, and *GCNT2*, were identified between group G-1 and group G-2, among which 209 were up-regulated, and 770 were down-regulated (Table S2). As illustrated in Figure 3B, clustering analysis indicated that the DEGs in the testicular tissues of Kazakh stallions at various ages demonstrated a consistent expression pattern, thereby reinforcing significant transcriptomic differences between the two sample groups.

### 3.5. GO Functional Annotation and KEGG Enrichment Analysis

**Figure 4:**
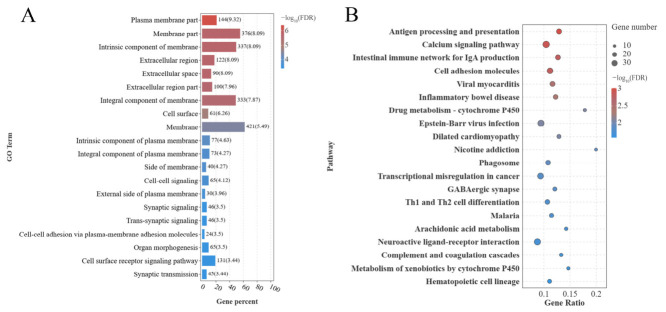
GO Annotation Terms and Results of KEGG Enrichment Analysis. (**A**) GO annotation terms for genes in Group G1 and Group G2; (**B**) KEGG enrichment analysis results for genes in Group G1 and Group G2. Note: (**B**) depicts the top 20 pathways with the lowest -log_10_(FDR). The vertical axis displays pathway names, and the horizontal axis shows gene ratios. Count values increase from left to right, with blue to red indicating progressively higher-log_10_(FDR).

GO annotation and KEGG pathway enrichment analysis were performed for DEGs between the G1 and G2 groups. As shown in Figure 4A, DEGs were predominantly associated with the following GO terms (Table S3): cell–cell signaling (BP), synaptic signaling (BP), and trans-synaptic signaling (BP); plasma membrane part (CC), membrane part (CC), and integral component of membrane (CC); and neurotransmitter receptor activity (MF), peptide receptor activity (MF), and channel activity (MF).

As illustrated in Figure 4B, KEGG enrichment analysis indicated the enrichment of DEGs in the Calcium signaling pathway, Cell adhesion molecules, and Neuroactive ligand–receptor interaction (Table S4).

### 3.6. Gene Set Enrichment Analysis

**Figure 5:**
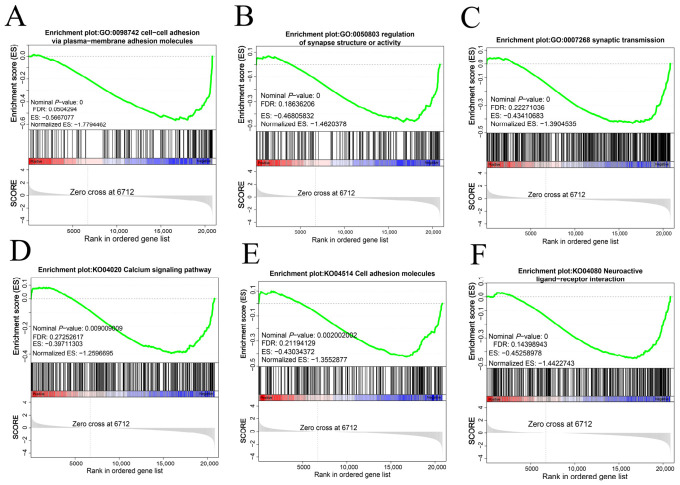
Gene set enrichment analysis results for testicular tissues from one- and two-year-old Kazakh horses.Note: Red (Positive end): represents the gene region associated with “high expression/positive correlation”; Blue (Negative end): represents the gene region associated with “low expression/negative correlation”.

Gene set enrichment analysis was performed on testicular transcriptome sequencing data of Kazakh horses at one and two years of age, with results consistent with the trends observed in GO and KEGG analyses. Significant enrichment was identified in biological processes such as cell–cell adhesion via plasma-membrane adhesion molecules, regulation of synapse structure or activity, and synaptic transmission. In KEGG analysis, signaling pathways including the Calcium signaling pathway, Cell adhesion molecules, and Neuroactive ligand–receptor interaction were significantly enriched (Figure 5).

### 3.7. PPI Network Analysis

**Figure 6:**
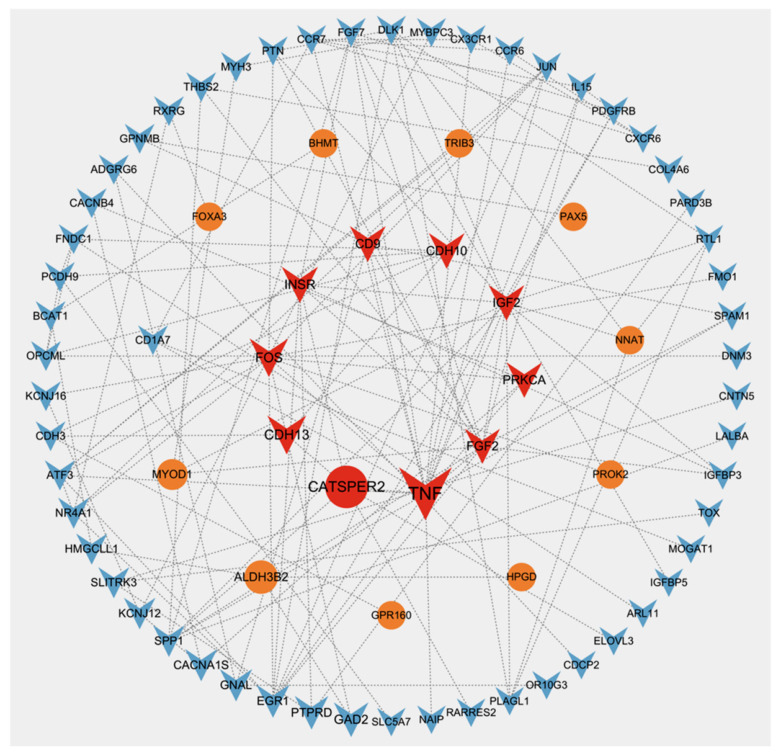
PPI network of DEGs and identification of core genes. Note: Circles represent up-regulated genes; triangles represent down-regulated genes.

In this study, a PPI network was constructed for DEGs in the testicular tissues of one- and two-year-old Kazakh horses. The identified core genes included *TNF*, *CATSPER2*, *CDH13*, *FOS*, and *CD9* (Figure 6).

### 3.8. RT-qPCR Analysis

**Figure 7:**
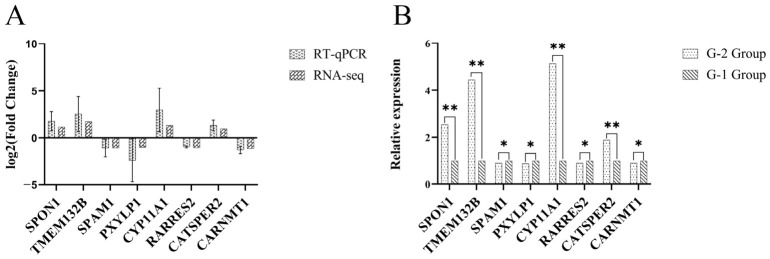
Validation of differentially expressed genes by RT-qPCR. (**A**) Log_2_(fold-change) comparison between RNA-seq and RT-qPCR for differentially expressed genes; (**B**) Relative expression of differentially expressed genes by RT-qPCR. Note: The asterisk (*) indicate significantdifferences (*p* < 0.05), while the double asterisk (**) indicate highly significant discrepancies (*p* < 0.01).

To verify the reliability of the RNA-seq data, based on differential expression significance (FDR value) and fold change in expression (log2FC), eight genes (*SPON1*, *TMEM132B*, *SPM1*, *PXYLP1*, *CYP11A1*, *RARRES2*, *CATSPER2*, *CARNMT1*) were selected from the DEGs for RT-qPCR validation. Expression trends for all genes were consistent between RT-qPCR and RNA-seq results, confirming the reliability of the identified sequencing data and expression profiles for subsequent analyses (Figure 7).

## 4. Discussion

The testis in the male animal reproductive system is crucial, and its development directly influences the male’s reproductive capacity. In this study, histological and morphological observations of the testes of one- and two-year-old Kazakh horses revealed that during the transition of the stallion testes from an immature to a mature state, the testicular tissue underwent changes oriented toward the formation of seminiferous tubules and initiated spermatogenesis. Zheng [23] conducted histomorphological analysis on testes collected from Duroc pigs from the newborn stage to sexual maturity, comprising nine different stages. Spermatogenic cells could be identified in the testes of 110-day-old boars, while round spermatids were found in 130-day-old testes, but spermatozoa and seminiferous tubule lumina were observed only in 150-day-old testes, with the diameter of the seminiferous tubules increasing after 30 days of age.

Subsequently, we conducted transcriptomic sequencing and analysis of testicular tissues from one- and two-year-old Kazakh horses. A total of 979 DEGs were identified, among which 209 were significantly up-regulated, and 770 were significantly down-regulated in the testes of two-year-old Kazakh horses. In this study, the *CD9* gene showed higher expression in the testicular tissue of 1-year-old horses. Previous studies have demonstrated that *CD9* is expressed in multiple testicular cell types and is involved in cell adhesion, migration, and signal transduction [24,25]. Its high expression pattern in immature testicular tissue suggests that *CD9* may be associated with specific states or interactions of germ cells or somatic cells in the early-stage testis, and may be involved in spermatogonial proliferation or adhesion processes [26,27]. Spermatogenesis is a strictly ordered and coordinated process, including three stages: differentiation of spermatogonial stem cells, meiosis of spermatocytes, and spermiogenesis. It is tightly regulated at the transcriptional, post-transcriptional, and translational levels [28]. In this study, high *CD9* gene expression in the testes of one-year-old Kazak horses suggests an accumulation of undifferentiated spermatogonia. *CYP11A1* is a protein located on the inner mitochondrial membrane and a marker gene for steroidogenic cells. Its abundance reflects the degree of differentiation of steroidogenic cells [29,30]. In this study, significantly upregulated expression of *CYP11A1* was detected in the testicular tissue of 2-year-old horses, suggesting that the steroidogenic activity of Leydig cells may be enhanced in this age group. This aligns with the biological expectation that testicular development at this stage requires higher testosterone levels to support sperm maturation [31]. The *CATSPER2* protein is part of the calcium-permeable *CATSPER* channel located in the main piece membrane of the sperm flagellum and is expressed exclusively in the testis during spermatogenesis [32]. The high expression of the *CATSPER2* gene in the testicular tissue of 2-year-old Kazakh horses corresponds to the transcriptomic features of active spermatogenesis and the potential for functional sperm production in sexually mature testes. This finding supports that 2 years of age represents an important time point for functional testicular development in Kazakh horses, though the quantitative relationship between its expression level and individual actual reproductive capacity requires further investigation. The DEGs identified in this study may play significant roles in testicular development and spermatogenesis, although the underlying mechanisms merit further investigation.

GO enrichment analysis of DEGs revealed their involvement in BPs associated with multicellular organism development. Bian Qiao et al. conducted transcriptomic sequencing on testicular tissues from DLY pigs in embryonic and adult stages. They found GO enrichment of DEGs concentrated in processes such as spermatogenesis, male gamete generation, and sexual reproduction [33]. KEGG enrichment analysis indicated that the up-regulated genes in the testes of two-year-old Kazakh horses were mainly enriched in signaling pathways, including the Calcium signaling pathway, Cell adhesion molecules, and Neuroactive ligand–receptor interaction. These pathways are associated with cell proliferation, hormone regulation, and sperm development. Notably, both GO and KEGG enrichment analyses identified multiple significant terms related to neuronal function and synaptic signaling. In testicular tissue, dense paracrine interactions and adhesive junctions exist among Sertoli cells within the seminiferous epithelium, various stages of germ cells, and interstitial cells, with similarities to signal processing in synapses. Therefore, these enrichment results are more likely to reflect intercellular communication and signal transduction during testicular development, crucial for the synchronization, differentiation of germ cells, and maintenance of microenvironmental homeostasis. Ca^2+^ participates in various cellular functions of testicular germ cells and somatic cells, particularly in modulating the response of the reproductive tract to endocrine hormones and local regulatory factors. Ca^2+^ transport across cell membranes may influence testicular development and steroid hormone synthesis [34,35]. In line with the findings of this study, Fang et al. [36] reported that genes highly expressed in mature yak testes were significantly enriched in the Calcium signaling pathway, highlighting the important and complex role of this pathway in testicular development and the maintenance of male traits. Cell adhesion molecules are critical components of intercellular adhesion structures that maintain normal connections between spermatogenic cells and Sertoli cells, among spermatogenic cells, and between Sertoli cells within the testicular tissue [37,38]. Cell adhesion molecules are cell-surface glycoproteins that mediate interactions between cells and other cells, as well as with the extracellular matrix. By facilitating adhesion and regulation of the activity of other membrane proteins, they modulate various cellular processes, including cell growth, proliferation, migration, and survival [39]. Xi et al. demonstrated that [40] DEGs during testicular development in hybrid sheep of different ages were significantly enriched in cell adhesion molecules. In studies on cryptorchidism in yaks, Yan et al. found that [41] the target genes corresponding to differentially expressed miRNAs were primarily involved in biological processes or pathways related to cell adhesion, suggesting that these factors may directly or indirectly influence the occurrence of cryptorchidism. Thus, DEGs enriched in the Calcium signaling pathway and Cell adhesion molecules in this study are essential for testicular development. The report on the transcriptome of Mongolian horse testes [13] compared the testes before and after sexual maturity; whereas in this study, by comparing two age points, we aimed to analyze the dynamic transcriptome changes specifically from pre-puberty to near sexual maturity. In terms of discoveries in core biological pathways, both studies showed consistency, identifying significant enrichment in pathways such as ‘Calcium signaling pathway’ and ‘Cell adhesion molecules’. Intercellular communication and calcium ion-mediated signal transduction are core molecular events in the testicular development process of equine species.

While this study systematically revealed the developmental characteristics and molecular regulatory mechanisms underlying Kazakh horse testis development during the critical sexual maturation period through histomorphology and transcriptome sequencing, and identified several key genes and pathways, some limitations remain to be addressed in future research. First, the sample size was relatively small, with only four horses in each age group. Although this meets the basic statistical requirements for exploratory RNA-seq studies and can identify candidate genes with high confidence, the limited sample size increases the risk of false negatives, indicating that subtle expression changes may not have been detected. Additionally, results from small samples are more susceptible to individual variations. Expanding the sample size in future studies would improve reliability. Second, the age points were limited to two (one and two years), failing to cover a continuous developmental timeline from the foal stage to adulthood, potentially overlooking dynamically regulated genes. Including multiple age points would provide a more comprehensive understanding of testicular development. Furthermore, the histological analysis was qualitative and lacked quantitative morphometric data such as seminiferous tubule diameter and interstitial area ratio, thereby limiting precise evaluation of structural changes. Transcriptome sequencing was performed on the whole testis tissue, containing a mixture of cell types, including seminiferous tubules, Leydig cells, and Sertoli cells. Thus, observed gene expression differences may reflect changes in cellular composition between age groups rather than true transcriptional alterations within specific cell types. The key DEGs and enriched pathways identified in this study should be regarded as preliminary candidates, as their specific biological functions and regulatory mechanisms have not been functionally validated.

In summary, the findings of this study provide a transcriptomic atlas and a set of candidate genes to better understand testicular development in Kazakh horses. However, the generalizability and precise regulatory roles of these candidates warrant further investigation through expanded sample sizes, integration of quantitative histology and hormone profiling, single-cell sequencing for cell-type resolution, and in-depth functional experiments.

## 5. Conclusions

We performed histomorphological observation and transcriptome sequencing analysis of testicular tissues from Kazakh horses at two different ages. Testicular tissue sections from one-year-old horses exhibited numerous seminiferous tubules with clearly visible density and an increased amount of interstitial tissue. In two-year-old horses, a complete lumen was formed within the tubules, with the epithelium consisting of one or multiple layers, and spermatogonia, spermatocytes, spermatids, and spermatozoa could be observed. DEGs were significantly enriched in the Calcium signaling pathway and Cell adhesion molecule pathway, suggesting their active roles. PPI network analysis identified key hub genes, including *TNF*, *CATSPER2*, *CDH13*, *FOS*, and *CD9*, thereby providing candidate genes for mechanistic investigation. Although the current findings are derived from comparisons of specific age groups and await functional validation, they establish a foundational molecular resource and generate testable hypotheses for understanding the genetic basis of reproductive development in Kazakh horses.

## Figures and Tables

**Table 1:** Overall detection of mRNA sequencing data.

Sample	Raw Data (bp)	Clean Data (bp)	Q20 (%)	Q30 (%)	GC (%)
G-1-1	12,685,166,100	12,534,409,823	98.80%	94.54%	50.09%
G-1-2	12,093,191,100	11,973,614,092	98.85%	94.67%	49.12%
G-1-3	12,272,421,600	12,106,261,510	98.81%	94.49%	49.86%
G-1-4	12,863,545,200	12,689,839,931	98.84%	94.75%	49.67%
G-2-1	14,968,677,600	14,760,442,787	98.74%	94.42%	51.67%
G-2-2	11,342,264,400	11,212,609,955	98.89%	94.73%	50.75%
G-2-3	12,872,604,900	12,714,955,206	98.71%	94.16%	51.66%
G-2-4	11,498,416,500	11,366,699,357	98.85%	94.67%	51.58%

Note: Samples: Sample name; Raw Data: Original sequencing data; Clean Data: Filtering of data; Q20: Proportion of bases with quality value greater than or equal to 20; Q30: Proportion of bases with quality value greater than or equal to 30; GC content: Calculation of the percentage of the total number of bases G and C to the total number of bases.

**Table 2:** Statistical analysis of reads aligned with the reference genome.

Sample	Total Reads	Total Mapped (%)	Unique Mapped (%)	Multiple Mapped (%)
G-1-1	83,502,734	79,611,080 (95.34%)	72,068,130 (86.31%)	7,542,950 (9.03%)
G-1-2	79,767,150	75,920,732 (95.18%)	70,493,494 (88.37%)	5,427,238 (6.80%)
G-1-3	80,865,568	77,135,591 (95.39%)	70,459,550 (87.13%)	6,676,041 (8.26%)
G-1-4	84,747,352	80,691,777 (95.21%)	73,805,008 (87.09%)	6,886,769 (8.13%)
G-2-1	98,520,126	92,631,207 (94.02%)	82,201,808 (83.44%)	10,429,399 (10.59%)
G-2-2	74,975,004	71,721,904 (95.66%)	64,831,387 (86.47%)	6,890,517 (9.19%)
G-2-3	84,912,482	81,116,168 (95.53%)	71,211,771 (83.86%)	9,904,397 (11.66%)
G-2-4	75,950,422	72,696,472 (95.72%)	63,176,235 (83.18%)	9,520,237 (12.53%)

Note: Samples: Sample name; Total reads: number of clean reads upon quality control; Total mapped: number (percentage) of reads aligned to the reference genome; Unique mapped: number (percentage) of reads aligned to a unique region of EquCab3.0 (subsequently analyzed for quantitation); Multiple mapped: number (percentage) of reads with alignment to many locations of EquCab3.0.

## Data Availability

The data presented in this study are openly available in BioProject with reference number PRJNA1377492.

## Supplementary Materials

The following supporting information can be downloaded at: https://www.mdpi.com/article/10.3390/ani16081220/s1, Table S1: Primers used in this study; Table S2: All differentially expressed genes; Table S3: GO enrichment analysis of differentially expressed mRNA. Table S4: KEGG pathway enrichment analysis of differentially expressed mRNA.
